# Estimating physical activity trends among blacks in the United States through examination of four national surveys

**DOI:** 10.3934/publichealth.2018.2.144

**Published:** 2018-05-29

**Authors:** Wanda M. Williams, Michelle M. Yore, Melicia C. Whitt-Glover

**Affiliations:** 1 School of Nursing, Rutgers University | Camden, Camden, NJ, USA; 2 Statistical Consultant, Orlando, FL, USA; 3 President & CEO, Gramercy Research Group, Winston-Salem, NC, USA

**Keywords:** African American, across the life span, secondary analysis, national datasets (BRFSS, NHANES, NHIS & YRBS), health behavior, health promotion, preventive measures

## Abstract

Physical activity is essential for overall good health and aids in the prevention and reduction of many diseases. In 2008, the U.S. Department of Health and Human Services (DHHS) issued the Physical Activity Guidelines for Americans to foster appropriate levels of physical activity at various ages of development. Despite these guidelines and the known benefit to being physically active; physical activity levels are significantly lower in Blacks, contributing to higher prevalence of poor health outcomes. Therefore, the purpose of this paper was to look at four national datasets [Youth Risk Behavior Survey (YRBS), Behavioral Risk Factor Surveillance System (BRFSS), The National Health and Nutrition Examination Survey (NHANES), and National Health Interview Survey (NHIS)] to identify any patterns and trends that could be used to improve physical activity behavior within this population. These national datasets were used to estimate the proportion of Black adults and youth meeting national physical activity recommendations overall—stratified by age, gender and other demographic characteristics, to help identify patterns. The proportion of Black youth reporting regular physical activity ranged from 33% to 52%; and of Black adults, 27% to 52%. Physical activity was highest among men, younger age groups, highest education and income groups, and those who were employed or married. Trends were consistent across surveys. Among Black youth, physical activity decline with increasing grade level, and improvements over the past 10 years have been minimal. The percentage of Black adults achieving physical activity guidelines has improved slightly over the last ten years, but physical activity participation is still low and continues to decline with age. Trends identified from examining these national datasets can be used to inform development of physical activity interventions aimed at promoting and maintaining regular physical activity behavior among high risk subgroups across the life span.

## 1. Introduction

Physical activity is essential for overall good health and aids in the prevention and reduction of many diseases. Physical activity has been defined as, “any bodily movement produced by the contraction of skeletal muscle that increases energy expenditure above a basal level” [1]; resulting in enhanced health, if done regularly. In 2008, the U.S. Department of Health and Human Services (DHHS) issued the *Physical Activity Guidelines* (PAG) for Americans to foster appropriate levels of physical activity at various ages of development, which would help ensure optimal level of health for all citizens [2]. The Physical Activity Guidelines recommend that children and adolescents should perform at least 60 minutes or more of physical activity daily. Adults and older adults should do at least 150 minutes of moderate intensity aerobic physical activity each week [2]. Being physically active is one of the most effective measures that Americans of all ages can do to help promote and maintain good health. Daily physical activity is recognized by DHHS as significant to improving a person's health and quality of life, and it is recognized by Healthy People 2020 (HP 2020) as a leading health indicator [3]. HP 2020 goals and objectives for physical activity represent the latest evidence aimed at individuals to help them meet physical activity guidelines.

Despite the known health benefits of daily physical activity, some population subgroups, including Blacks, consistently fall short of meeting national *Physical Activity Guidelines* [4]. Given the high prevalence of poor health outcomes associated with low levels of physical activity among Blacks, understanding patterns of physical activity among this population subgroup is important for identifying targeted strategies to promote physical activity within population subcategories. Therefore, the purpose of this paper was to look at four national datasets [Youth Risk Behavior Survey (YRBS), Behavioral Risk Factor Surveillance System (BRFSS), The National Health and Nutrition Examination Survey (NHANES), and National Health Interview Survey (NHIS)] to identify any patterns and trends that could be used to improve physical activity behavior within this population.

## 2. Methods

Four national datasets (YRBS-2015; BRFSS-2015; NHANES, 2011–2014; and NHIS) were used to estimate the proportion of Black adults and youth meeting national physical activity recommendations overall—stratified by age, gender and other demographic characteristics, to help identify patterns. These surveys use different survey methods, questionnaire styles, and question formats to inquire about respondents' physical activity levels, which allows for an in-depth comparison of physical activity measurements. Compiling and evaluating data from multiple survey sources allowed for a more complete overall view of the physical activity levels and trends among Blacks than evaluating results from a single survey. Comparing results from these data sources will give a broader picture of physical activity levels.

These national surveys are supported by the U.S. Department of Health and Human Services (DHHS) and have been approved by the NCHS Research Ethics Review Board (ERB) and the Office of the IRB (OIRB), depending on the survey [5]. A brief overview of each survey is included in Table 1, with more details below regarding how physical activity guidelines were measured for each dataset. For each dataset, meeting regular physical activity requirements was defined as meeting the aforementioned 2008 PAG guidelines [2].

**YRBS** is one of the six categories under the Youth Risk Behavior Surveillance System (YRBSS), which monitors health-risk behaviors that contribute to the leading causes of death and disability among youth and young adults [6]. YRBS is a national school-based survey that is representative of all public and private high schools (9th–12th grades), conducted by the CDC bi-annually in all 50 states and the District of Columbia. To ensure a nationally representative sample of students in grades 9 to 12, a three-stage cluster sample design is utilized [6]. The 2015 YRBS sample size for Black students is 1,667. Youth physical activity levels were ascertained from the Youth Risk Behavior Survey 2015 (YRBS) [6]. Students were asked “During the past 7 days, on how many days were you physically active for a total of at least 60 minutes per day? (Add up all the time you spent in any kind of physical activity that increased your heart rate and made you breathe hard some of the time)” [6]. Meeting physical activity recommendations for youth was defined as being physically active for at least 60 minutes on 5 or more days per week.

**BRFSS** is the nation's premier system of health-related telephone surveys that collects data in all 50 states as well as the District of Columbia and three U.S. territories regarding their health-related risk behaviors, chronic health conditions and use of preventive services [7]. The 2015 BRFFS sample size for Black adults was 34,346. Questions regarding physical activity for BRFSS were asked on odd years [7]. The BRFSS questionnaire asked respondents if they participated in physical activity outside of work. If the respondent answered yes, they were asked to identify the top 2 activities and the number of times per week or month, and the number of hours or minutes they typically did the activity. These activities were converted to metabolic equivalent (MET) levels and classified as moderate- or vigorous-intensity. The number of daily minutes at each intensity level was used to determine achievement of PAG recommendations.

**NHANES** is a national survey that combines one-on-one interviews with physical and laboratory examinations to assess the health and nutritional status of adults and children in the United States [5]. It is conducted annually and is representative of the country. The health interviews are conducted within a person's home, with medical examinations carried out in specially designed and equipped mobile examination centers [5]. A random subsample of NHANES participants were selected to be included in the medical examination. During the exam, their height and weight were measured, and used to calculate BMI. The 2011–2014 NHANES sample size for Black adults was 2,809. NHANES data asked physical activity questions in the personal interview section of the survey. The questions inquired whether the respondents participated in vigorous recreational activity, and if they answered yes, they were asked the number of days and minutes per day they performed the activity. The same questions were asked for moderate-intensity activity. A person was classified as meeting the recommended level of physical activity if participation included at least 150 minutes per week of moderate-intensity activity, or at least 75 minutes per week of vigorous- intensity activity, or a combination of at least 150 minutes of moderate- and vigorous-intensity activity. NHANES analyses were weighted using the interview weight. Because BMI measurements were for a subsample of the participants, BMI analyses were weighted using the exam weight. Although NHANES collects objective data of physical activity with use of accelerometers, objective measures were not considered for the current analysis [8].

**NHIS** is a cross-sectional household interview survey conducted by the National Center for Health Statistics (NCHS) [9]. It is an on-going survey that is updated every 10 years and drawn from each State and the District of Columbia. NHIS data is used to monitor trends in illness and disability and to track progress toward achieving national health objectives [9]. NHIS sample size for Black Adults was 5,057 in 2014. The NHIS physical activity questions were asked in the Sample Adult module. Respondents were asked how often they performed vigorous leisure-time activities that caused large increases in breathing or heart rate, and the length of time they performed these activities. Respondents were also asked how often they performed light or moderate leisure-time activities, and the length of time they did these activities. Achievement of PAG recommendations was classified as described above.

### Statistical analysis

Each survey used a complex, sample design, which required weighted data analysis to calculate valid estimates of the population. NHIS used a complex, multi-stage sample design; which also included clustering, stratification, and over-sampling of specific populations. BRFSS uses a state-based, stratified random sample of both landline and cellular telephones. NHANES used a complex, multi-stage, probability sampling design with oversampling of certain groups (which changes with new survey cycles).

In an unweighted data analysis, each record has the same weight. Because of the survey designs, each survey had specific sampling weights which allow calculations to be generalizable to the larger population(s) and to enable appropriate calculations of variance according to the specific study design. STATA software [10] was used to incorporate the proper sample weights for variance estimation. As this paper was intended to be a descriptive analysis, physical activity prevalence estimates and 95% confidence intervals were calculated to indicate the percentage of people meeting the physical activity recommendations, overall and within population subgroups.

**Table 1.** Overview of National Data Sets.

Survey	Mode of Data Collection	Target Population	Total Sample Size	Conducted	Question to assess PA
Youth Risk Behavior Survey (YRBS), 2015	Questionnaires administered in the classroom	High school students (9^th^–12^th^ grade)	1667	Biennially	Consists of five PA questions; two questions are directly related to PA regarding frequency (times/day): During the past 7 days, on how many days were you physically active for a total of at least 60 minutes per day? (Add up all the time you spent in any kind of physical activity that increased your heart rate and made you breathe hard some of the time.). One question is related to time spent in physical education (PE) classes and a question about involvement with a sport or a team
Behavioral Risk Factor Surveillance System (BRFSS). 2005 [*]	Telephone	Adults only	34,346	Annually	1. During the past month, other than your regular job, did you participate in any physical activities or exercises such as running, calisthenics, golf, gardening, or walking for exercise? 2. What type of PA or exercise did you spend the most time doing during the past month? 3. How many times per week or per month did you take part in this activity during the past month? 4. And when you took part in this activity, for how many minutes or hours did you usually keep at it? 5. What other type of physical activity gave you the next most exercise during the past month? 6. How many times per week or per month did you take part in this activity during the past month? 7. And when you took part in this activity, for how many minutes or hours did you usually keep at it? 8. During the past month, how many times per week or per month did you do physical activities or exercises to strengthen your muscles?
National Health and Nutrition Examination Survey (NHANES), 2011–2014	Face-to-face interviews in homes and mobile centers (labs and objective monitors)	Adults & Children	2809	Annually	16 questions aimed at assessing PA and fitness. A sample question from the NHANES survey - During the past 7 days, on how many days were physically active for a total of at least 60 minutes per day? Add up all the time you spent in any kind of physical activity that increased your heart rate and made you breathe hard some of the time?
The National Health Interview Survey (NHIS), 2014	Face-to-face interviews	Adults & Children	5057	Annually	Includes a wide range of questions regarding PA from type and frequency of PA, for example, walking and for how long. Questions are asked about membership to fitness facilities and participation with sports and other type of exercises, as well as availability and access to parks or recreational areas. There is also a question regarding a doctor or other health professional recommending any type of exercise or PA, and if so what and how often?

PA: Physical Activity; All surveys are collected in the US & DC; [*] = as well as the territories.

## 3. Results

An overview of participant characteristics and physical activity prevalence estimates is included in Table 2 for youth and Table 3 for adults. The YRBS shows that, overall, less than half of Black youth report meeting PAG recommendations. As evidenced by non-overlapping confidence intervals, boys reported significantly more physical activity than girls. Among both boys and girls, physical activity significantly declined by 12^th^ grade (proxy for increasing age), and also declined with increasing body mass index levels.

Data for adults from all sources (BRFSS, NHANES & NHIS) showed that significantly higher percentages of men (45% to 52%) compared to women (27% to 41%) reported meeting PAG recommendations. Both men and women showed a significant decline in physical activity as they aged; for example, NHANES data show that the percentage of physical activity in men ages 25–44 was 66% and declined to 22% by age ≥ 65; and among women the decline was from 34% (ages 25–44) to 18% (age ≥ 65). Both men and women with higher educational levels reported higher percentages of physical activity; higher education usually equates to higher income levels, and as income levels increased, so did physical activity participation for both men and women. Participants who *“were employed”* were more physically active across all data sources and gender groups, although physical activity data on these surveys was assessing leisure time physical activity; inclusion of work-related physical activity by some participants cannot be ruled out. *“Never married men”* reported higher percentages (49% to 57%) of physical activity across all three datasets versus *“married men”* (39% to 49%). BRFSS and NHIS data showed that women who were *“married/living with a partner”* reported higher percentage (42%) of physical activity participation than women *“No longer married,”* or *“Never married”* (24% to 28%).

NHANES data stratified by weight status showed a steady decline in the proportion of Black women meeting national physical activity recommendations as weight status increased; normal weight (< 25.0 kg/m^2^) 34% compared to obese (25.0–29.9 kg/m^2^) 29%, and severely obese (≥ 30.0 kg/m^2^) women at 24%. Differences in physical activity participation by weight status were not as apparent in men.

Regional data from NHIS show that the Northeast region (Connecticut, Delaware, Maine, Massachusetts, New Hampshire, New Jersey, New York, Pennsylvania, Rhode Island and Vermont) reported lower percentages (35% to 38%) of adults being physically active. The western region (Mountain States: Montana, Wyoming, Colorado, New Mexico, Idaho, Utah, Arizona, and Nevada); and Pacific States (Washington, Oregon, California, Alaska, and Hawaii) reported higher percentages (≥ 50%) of adults being physical activity.

**Table 2.** Percentage of Black Children in the United States (Grades 9^th^–12^th^) Meeting ≥ 60 Minutes of Physical Activity on 5 or more days/week.

		Total Black Children					
		Sampled (N = 1,667)		Boys (n = 837)		Girls (n = 821)	
		43.4	(38.8–48.1)	52.2	(46.1, 58.3)	33.4	(28.1, 38.6)
*Grade*	*N*	*% by Grade meeting PA Levels*					
9	424	47.5	(38.0, 57.0)	56.6	(45.5, 67.7)	35.3	(26.1, 44.6)
10	424	44.5	(39.4, 49.7)	51.8	(44.3, 59.3)	37.2	(27.8, 46.7)
11	423	46.1	(37.7, 54.5)	54.9	(41.0, 68.7)	35.5	(26.7, 44.3)
12	387	34.1	(27.7, 40.5)	44.0	(33.3, 54.6)	24.2	(18.6, 29.8)
*Body Mass Index (BMI)*	*N*	*% by BMI meeting PA Levels*					
Normal weight	986	47.0	(42.5, 51.5)	57.3	(50.8, 63.7)	35.2	(29.2, 41.1)
Overweight	242	41.5	(33.0, 49.9)	55.7	(44.5, 66.8)	31.1	(20.3, 42.0)
Obese	256	38.4	(25.5, 51.3)	43.2	(23.9, 62.4)	31.9	(18.2, 45.7)

Source: Youth Risk Behavior Survey (**YRBS**)-2015; PA: Physical Activity.

**Table 3.** Overview of Physical Activity Prevalence among Black Adults in the US from National Surveys.

	Total - Estimate % (95% CI)			Men - Estimate % (95% CI)			Women - Estimate % (95% CI)		
	BRFSS	NHANES	NHIS	BRFSS	NHANES	NHIS	BRFSS	NHANES	NHIS
**Sample size N**	34,346	2,809	5,057	n = 12,394	n = 1354	n = 2047	n = 21,952	n = 1455	n = 3010
**Overall Pysical Activity**	—	—	—	47.7 (46.0–49.3)	44.6 (41.6–47.6)	51.6 (48.6–54.6)	40.7 (39.4–42.1)	27.4 (23.8–31.2)	38.2 (35.7–40.7)
**Age**									
18–24	2,164 (6.3%)	457 (16.3%)	472 (9.3%)	56.3 (51.4–61.2)	66.4 (60.2–72.7)	61.9 (52.8–70.9)	46.4 (41.8–51.1)	34.3 (23.6–45.1)	37.2 (29.1–45.3)
25–44	8,349 (24.3%)	795 (28.3%)	1,714 (33.9%)	46.8 (43.8–49.7)	54.8 (49.0–60.6)	62.3 (57.3–67.3)	41.2 (38.8–43.6)	29.9 (25.3–34.6)	45.0 (41.2–48.7)
45–64	13,690 (39.9%)	1,011 (36.0%)	1,838 (36.3%)	44.6 (41.9–47.3)	31.8 (27.3–36.2)	41.4 (37.1–45.8)	39.0 (36.9–41.2)	25.0 (19.2–30.8)	38.8 (35.0–42.6)
65+	9,669 (28.2%)	546 (19.4%)	1,033 (20.4%)	49.4 (45.8–53.0)	21.9 (17.5–26.3)	33.7 (27.8–39.5)	38.9 (36.4–41.4)	17.6 (12.8–22.5)	21.5 (17.6–25.5)
**Education**									
< HIgh School	4,243 (12.4%)	578 (20.6%)	—	42.7 (38.0–47.4)	26.4 (20.0–32.7)	not available	28.2 (24.7–31.6)	12.3 (6.9–17.7)	not available
High School Graduate	11,282(32.8%)	703 (25.0%)	—	45.4 (42.6–48.1)	38.5 (32.7–44.4)	not available	37.4 (34.9–39.9)	21.2 (16.5–25.8)	not available
Some College	9,488 (27.6%)	885 (31.5%)	—	50.3 (47.2–53.5)	50.3 (44.1–56.6)	not available	43.8 (41.3–46.2)	28.4 (23.4–33.3)	not available
College Graduate	9,189 (26.8%)	459 (16.3%)	—	51.6 (48.5–54.8)	61.4 (52.5–70.2)	not available	48.7 (46.2–51.2)	41.5 (36.9–46.1)	not available
**Income (BRFSS)**									
< $15 ,000	5,629 (16.4%)	—	—	42.1 (37.6–46.5)	—	—	36.6 (33.5–39.8)	—	—
$15,000–$25,000	6,901 (20.1%)	—	—	46.2 (42.4–50.0)	—	—	36.9 (34.2–39.7)	—	—
$25,000–$35,000	3,528 (10.3%)	—	—	47.9 (42.3–53.4)	—	—	40.2 (36.1–44.3)	—	—
$35,000–$50,000	3,845 (11.2%)	—	—	47.7 (43.0–52.3)	—	—	44.8 (40.4–49.1)	—	—
≥ $50,000	8,180 (23.8%)	—	—	50.9 (47.9–53.8)	—	—	47.7 (44.9–50.5)	—	—
**Income (NHIS - NHANES)**									
$0–$34,999	—	1,423 (50.7%)	2,476 (49.0%)	—	39.2 (34.9–43.5)	43.2 (40.0–46.4)	—	23.4 (19.0–27.7)	31.0 (28.9–33.2)
$35,000–$74,999	—	684 (24.4%)	1,206 (23.8%)	—	47.9 (41.4–54.3)	51.2 (47.0–55.3)	—	31.6 (27.3–35.9)	43.7 (39.9–47.6)
$75,000–$99,999	—	204 (7.3%)	318 (6.3%)	—	53.1 (41.3–64.8)	55.9 (48.2–63.6)	—	37.3 (23.5–51.2)	44.4 (36.5–52.3)
$100,000+	—	274 (9.8%)	371 (7.3%)	—	52.4 (44.5–60.4)	66.5 (59.7–73.3)	—	35.2 (28.6–41.9)	54.7 (47.4–62.0)
**Employment Status**									
Employed	15,638 (45.5%)	1,405 (50.0%)	3,013 (59.6%)	48.7 (46.5–51.0)	47.6 (43.9–51.3)	58.0 (54.3–61.7)	42.5 (40.6–44.4)	30.9 (26.7–35.0)	45.0 (41.6–48.4)
Not Employed	18,708 (54.5%)	1,402 (49.9%)	2,044 (40.4%)	46.2 (43.7–48.7)	40.5 (35.2–45.8)	39.7 (35.1–44.3)	38.8 (36.9–40.7)	23.3 (18.5–28.0)	27.4 (23.8–31.0)
**Marital Status**									
Married/Living With	11,591 (33.7%)	1,086 (38.7%)	358 (7.1%)	49.3 (46.7–51.8)	39.3 (33.6–45.0)	45.3 (35.1–55.6)	41.8 (39.4–44.2)	25.7 (19.5–32.0)	42.0 (33.4–50.7)
No longer married	12,261 (35.7%)	713 (25.4%)	2,757 (54.5%)	41.1 (37.7–44.5)	31.7 (26.5–36.9)	49.0 (45.4–52.6)	37.8 (35.7–39.9)	23.9 (19.2–28.6)	38.4 (35.3–41.5)
Never married	10,262 (29.9%)	831 (29.6%)	1,921 (38.0%)	49.1 (46.3–52.0)	55.1 (50.3–60.0)	57.3 (52.2–62.4)	41.8 (39.5–44.2)	29.4 (25.2–33.6)	37.3 (32.9–41.7)
**Body Mass Index (BMI)**									
< 25.0 kg/m^2^	7,506 (21.9%)	704 (25.1%)	1,291 (25.5%)	49.8 (46.6–53.0)	46.0 (41.1–50.9)	53.5 (47.6–59.4)	45.2 (42.2–48.3)	34.0 (27.4–40.7)	37.5 (32.2–42.8)
25.0–29.9 kg/m^2^	10,853 (31.6%)	758 (27.0%)	1,592 (31.5%)	49.5 (46.7–52.4)	47.3 (42.3–52.3)	53.1 (48.7–57.5)	45.7 (43.2–48.2)	28.9 (22.6–35.1)	46.3 (41.3–51.3)
≥ 30.0 kg/m2	12,879 (37.5%)	1,224 (43.6%)	1,998 (39.5%)	44.4 (41.6–47.2)	41.0 (34.8–47.1)	48.8 (43.7–53.8)	36.6 (34.5–38.7)	24.0 (21.2–26.9)	34.3 (31.0–37.6)
**Region of U.S.**									
Northeast	—	—	680 (13.4%)	—	—	38.4 (29.7–47.1)	—	—	34.8 (28.4–41.)
Midwest	—	—	864 (17.1%)	—	—	54.2 (47.1–61.3)	—	—	34.3 (28.2–40.4)
South	—	—	3,092 (61.1%)	—	—	54.5 (50.6–58.4)	—	—	38.7 (35.4–41.9)
West	—	—	421 (8.3%)	—	—	50.7 (41.5–60.0)	—	—	50.8 (41.2–60.3)

Sources: Behavioral Risk Factor Surveillance System **(BRFSS**), 2015; The National Health and Nutrition Examination Survey (**NHANES**), 2011–2014; & National Health Interview Survey (**NHIS**), 2014.

## 4. Discussion

The percentage of Black adults achieving physical activity guidelines has improved slightly over the last ten years, but physical activity participation is still low and continues to decline with age. Physical activity is a key factor for healthy aging; however, trends noted in this analysis showed that physical activity declines with age, beginning in youth. The current analysis shows that since 2013 there has been little or no detectable change in the proportion of students in grades 9 to 12 who met the physical activity guidelines for aerobic physical activity [11]. The targeted goal of 32% for adolescents meeting aerobic physical activity guidelines for HP 2020 at midcourse review has not been met [11]. Most programs aimed at youth are school-based, which are viewed as more universally accessible. Yet effective school-based programs are not readily available or accessible, especially to Black youth in low income communities [12]. A study by Sutherland et al. conducted in 2016 looked at the cost effectiveness of a multi-component intervention aimed at improving physical activity in adolescents in secondary schools located in low-income communities, and found them to be cost-effective [13]. The Physical Activity 4 Everyone (PA4E1) program, evaluated by Sutherland et al., may be one good example of a program that could be implemented more universally [13]. Data from the current analysis continue to show declines in physical activity as age increases among adults. Given the studies supporting the positive impact of regular physical activity in older adults, which can significantly reduce health problems associated with heart disease, arthritis and diabetes [14],[15], studies are needed to understand how to begin physical activity at young ages and continue it across the lifespan.

Higher prevalence of regular physical activity was observed among boys/men compared with girls/women. This finding is consistent with the literature indicating a significant difference in physical activity levels between males and females regardless of age [16],[17]. A study by Lenhart et al. showed that girls were less likely to be active than boys; and girls required more structured physical activity events than boys [18]. These differences noted in childhood continue into adulthood, with women reporting less physical activity than men. Factors that may influence higher levels of physical activity among boys/men compared with girls/women include perceptions of safety, concerns about personal appearance, cultural attitudes about the appropriateness of physical activity for boys/men *vs.* girls/women, and caregiving duties associated with girls/women that might preclude participation in leisure-time physical activity [17],[19],[20]. Based on data from the current analysis, only 27% to 41% of Black women are meeting the target physical activity goal of 48 % set by HP 2020, indicating significant improvement is needed.

The data showed that Black women who were *“married/living with a partner”* reported a higher percentage of physical activity participation than women *“No longer married”* or *“Never married”.* Studies show that Black women with a supportive partner who offers encouragement and financial support tend to engage in regular physical activity [21],[22]. In contrast, single Black women are less physically active, which may be due to their increased family responsibilities [23]: Black women tend to be the main caregivers for children and others in the household, and therefore may have less time to engage in physical activity [24],[25]. Cultural considerations may also prohibit some women from engaging in physical activity [26],[27].

Consistent with the previous study, individuals with higher education and income (regardless of gender) also had higher prevalence of being physical activity [28]. Studies support that income level does have a bearing on physical activity [24],[29]. A study by Van Domelen et al. in 2011 revealed that men employed full-time were more physically active [30], a finding that was also consistent with this analysis. Sun and colleagues revealed that higher socioeconomic status was positively associated with young Black women being physically active [31], suggesting that increased consideration of income status when designing physical activity programs may be warranted.

Regional data from NHIS show that the Northeast reported lower percentages of adults being physically active. The Western region and Pacific states reported higher percentages of adult being physical activity. The regions that are less physically active coincide with higher chronic disease indicators (diabetes, heart disease and obesity) [32]; supporting the importance of being physically active to help reduce chronic diseases.

## 5. Limitations and strengths

Some limitations should be considered when interpreting multiply surveys. For example, the data collection processes varied from phone surveys, to one-on-one interviews, to questionnaires, thus contributing to differences in sample selection, participant responses and results. Likewise, all three-national datasets rely on self-reported measures of physical activity, which is subject to recall bias, and over or under reporting. Additionally, leisure-time physical activity is the primary focus of the national surveillance systems, which overlooks other forms or domains of physical activity (i.e., household, transportation, or occupational). Assessment of all domains of physical activity is needed to provide true estimates of overall physical activity prevalence and to determine if other domains of physical activity offer health benefits. Strengths of the current analysis include samples from national datasets, large sample sizes with which to draw point estimates, and consistency of the trends described in this paper.

## 6. Conclusions

It is well established that physical inactivity is not good for a person's health, and some physical activity is better than none. The benefits of regular physical activity in preventing and reducing risks for several chronic conditions, such as cardiovascular disease, hypertension, diabetes, obesity, and some forms of cancers, are well documented. Measures to promote regular daily physical activity are essential for reducing these risks and understanding gender and age difference would aid in the development of effective and sustainable physical activity programs. All people, regardless of age, can benefit from some form of daily physical activity, and older adults >65 years should continue to be as physically active as their abilities and conditions will allow.

Statistics indicate that Blacks are less physically active than Whites across the lifespan [4],[6]. Examining national datasets helped identify trends that could be used to inform development of interventions aimed at promoting and maintaining regular physical activity among Blacks across generations and subgroups (women, youth and elderly). Although objective measures are now available from NHANES data, other objective data sources are also needed to obtain and compare more accurate and specific information regarding physical activity behavior and patterns, especially in racial/ethnic minority groups. Based on HP 2020 midcourse review some progress has been made in meeting physical activity guidelines; however, the results from this analysis suggest that more research is needed to gain an in-depth understanding of the psychosocial and economic factors that influence Blacks' participation in physical activity. Obtaining this information could help with developing and implementing effective preventive interventions aimed at getting people of all ages moving to a healthier lifestyle.
